# TIA-1 Cytotoxic Granule-Associated RNA Binding Protein Improves the Prognostic Performance of CD8 in Mismatch Repair-Proficient Colorectal Cancer

**DOI:** 10.1371/journal.pone.0014282

**Published:** 2010-12-10

**Authors:** Inti Zlobec, Eva Karamitopoulou, Luigi Terracciano, Salvatore Piscuoglio, Giandomenica Iezzi, Manuele Giuseppe Muraro, Giulio Spagnoli, Kristi Baker, Alexandar Tzankov, Alessandro Lugli

**Affiliations:** 1 Institute for Pathology, University of Basel, Basel, Switzerland; 2 Second Department of Pathology, Attikon University Hospital, Athens, Greece; 3 Research Group Human Genetics, Department of Biomedicine, University of Basel, Basel, Switzerland; 4 Institute for Surgical Research and Hospital Management, University of Basel, Basel, Switzerland; 5 Department of Gastroenterology, Brigham and Women's Hospital, Boston, Massachusetts, United States of America; University of Barcelona, Spain

## Abstract

**Background:**

Evidence suggests a confounding effect of mismatch repair (MMR) status on immune response in colorectal cancer. The identification of innate and adaptive immune cells, that can complement the established prognostic effect of CD8 in MMR-proficient colorectal cancers patients, representing 85% of all cases, has not been performed.

**Methodology/Principal Findings:**

Colorectal cancers from a test (n = 1197) and external validation (n = 209) cohort of MMR-proficient colorectal cancers were mounted onto single and multiple punch tissue microarrays. Immunohistochemical quantification (score 0-3) was performed for CD3, CD4, CD8, CD45RO, CD68, CD163, FoxP3, GranzymeB, iNOS, mast cell tryptase, MUM1, PD1 and TIA-1 tumor-infiltrating (TILs) reactive cells. Coexpression experiments on fresh colorectal cancer specimens using specific cell population markers were performed. In the test group, higher numbers of CD3+ (p<0.001), CD4+ (p = 0.029), CD8+ (p<0.001), CD45RO+ (p = 0.048), FoxP3+ (p<0.001), GranzymeB+ (p<0.001), iNOS+ (p = 0.035), MUM1+ (p = 0.014), PD1+ (p = 0.034) and TIA-1+ TILs (p<0.001) were linked to favourable outcome. Adjusting for age, gender, TNM stage and post-operative therapy, higher CD8+ (p<0.001; HR (95%CI): 0.66 (0.64-0.68)) and TIA-1+ (p<0.001; HR (95%CI): 0.56 (0.5-0.6)) were independent prognostic factors. Moreover, among patients with CD8+ infiltrates, TIA-1 further stratified 355 (35.6%) patients into prognostic subgroups (p<0.001; HR (95%CI): 0.89 (95%CI: 0.8-0.9)). Results were confirmed on the validation cohort (p = 0.006). TIA-1+ cells were mostly CD8+ (57%), but also stained for TCRγδ (22%), CD66b (13%) and only rarely for CD4+, macrophage and NK cell markers.

**Conclusions:**

TIA-1 adds prognostic information to TNM stage and adjuvant therapy in MMR-proficient colorectal cancer patients. The prognostic effect of CD8+ TILs is confounded by the presence of TIA-1+ which translates into improved risk stratification for approximately 35% of all patients with MMR-proficient colorectal cancers.

## Introduction

The prognosis of patients with colorectal cancer can be viewed as an interaction between tumor- and host-related factors [1]. Experimental evidence over the last 20 years provides support for the concept of immunosurveilllance in cancer, implicating both innate and adaptive immune responses [2]. In colorectal cancer, CD3, CD4, CD8, CD20, Granzyme B, and FoxP3+ tumor infiltrating lymphocytes (TILs) have been identified as potential indicators of outcome, while the identification of cytotoxic T-lymphocytes, mast cells and dendritic cells as elicitors of anti-tumor responses has underlined new avenues for potential immunotherapies [3], [4], [5].

In colorectal cancer, the balance between host- and tumor-related factors is exemplified by mismatch repair (MMR)-deficient sporadic cases, accounting for approximately 15% of all tumors and characterized by defective MMR machinery [6]. Patients with these MMR-deficient cancers are described as having abundant CD8+ cytotoxic T-cell infiltrate [7] and are often linked to more “favourable” tumor-related features, namely the presence of a pushing tumor border configuration, the presence of a distinctive band of peritumoral lymphocytic inflammation and little tumor budding, the latter a histomorphological hallmark of epithelial mesenchymal transition [8]. This phenotypic constellation, by “tipping the scale” in favour of a strong defence may in part be responsible for the more favourable overall prognosis of patients with MMR-deficient compared to MMR-proficient tumors [9]. Interestingly, despite the known confounding effect of MMR status on immunological responses in colorectal cancer, comprehensive analyses of cell types involved in immune response and inflammation have not yet been systematically performed for MMR-proficient cancers, encompassing 85% of all colorectal cancer cases.

Therefore, the aim of this study was to determine the additional prognostic benefit, over the performance of CD8 alone, of 12 tissue-based immunologic biomarkers in MMR-proficient colorectal cancer patients. These protein markers including CD3, CD4, CD45RO, CD68, CD163, FoxP3, GranzymeB, iNOS, mast cell tryptase, MUM1p, PD1 and TIA-1 were specifically selected to cover the widest range of possible cell types involved in innate and adaptive immune responses. This was accomplished using an initial test group of 1197 patients and an external validation cohort of 209 patients, respectively with complete clinico-pathological and follow-up data.

## Methods

### Ethics Statement

Written consent has been given from the patients for their information to be stored in the hospital database and used for research**.** The use of tissue was approved by the corresponding Ethics Committees of the University Hospital of Basel and University of Athens. Freshly excised clinical specimens included in this study were collected from consenting patients undergoing surgical treatment at Basel University Hospital.

### Test Group

#### Patients and specimen characteristics

1420 primary pre-operatively untreated, unselected sporadic colorectal cancer patients treated at the University Hospital of Basel between the years 1987 and 1996 were included in this study. Haematoxylin and eosin (H&E) stained slides were reviewed by an experienced gastrointestinal pathologist (L.T.) and clinical data were retrieved from patient records, where available. Clinical outcome of interest was cancer-specific survival time. A tissue microarray of these 1420 patients was constructed. From each patient, one representative tumor block was punched using a tissue cylinder 0.6 mm in diameter. Tissue was brought into one recipient paraffin block (3×2.5 cm) using a homemade semi-automated tissue arrayer. 57 tissues from normal colorectal mucosa were included as a control.

#### Assay Methods

The tissue microarray was immunostained for 13 immunological protein markers and mismatch repair markers. Protocols for MLH1, MSH2, MSH6, CD8 and FoxP3 have been described elsewhere [10]. Briefly, the remaining protocols were carried out as follow: CD163; NeoMarkers, MS-1103, monoclonal, 1∶40, Citrate buffer pH6, 100°C, 30′; CD20; Dako, M0755, monoclonal, 1∶50 Citrate buffer pH6, 100°C, 30′; CD4; NeoMarkers, MS-1528, monoclonal, 1∶40, Citrate buffer pH6, 100°C, 60′; CD68; Dako, M0876, monoclonal, 1∶200, Citrate buffer pH6, 100°C, 15′; GranzymeB; Novocastra, NCL-L-GRAN-B, 1∶10, Citrate buffer pH6, 120°C, 10′, iNos; Abcam, ab15323, polyclonal, 1∶100 ER1 buffer, 20′, Mast cell tryptase; Dako, M7052, monoclonal, 1∶2000, no retrieval; Mum1; Dako, M7259, monoclonal, 1∶50, Citrate buffer pH6, 100°C, 30′; PD1; R&D Systems, AF1086, monoclonal 1∶40 Citrate buffer pH6, 120°C, 10′; TIA-1; Immunotech, IM2550, monoclonal, 1∶250, no retrieval; CD3; Dako, monoclonal, Citrate buffer; 1∶50; and CD45RO; Thermo Scientific UCHL-1, monoclonal, Citrate buffer, 1∶500. Negative control tissues were tested and underwent the same protocol with the primary antibody omitted.

Protein markers were scored by three observers (A.L., S.P., A.T.) by analyzing the number of positive cells per tissue microarray punch. No image analysis software was used. The total number of immunoreactive cells within the tumor microenvironment was evaluated, independent of localization (intratumoral or stromal). The total number of cells per tissue microarray punch were given scores of 0 when no positive cells were present, and scores 1, 2 and 3 when 1-10 positive cells, 11-50 positive cells and >50 positive cells per punch could be observed, respectively. For PD1 and iNOS, cases were scored as the complete absence or presence of any positive cells.

### Validation Group

221 unselected, non-consecutive colorectal cancer patients treated at the Attikon University Hospital, University of Athens, Greece between the years 2004 and 2006 were included as an independent validation group. H&E slides were reviewed and clinical data retrieved from patient records (**Table 1**). Clinical outcome of interest was cancer-specific survival time. A tissue microarray of primary colorectal cancer resections from these 221 patients was constructed. In order to exclude bias due to possible tumor heterogeneity, each patient had multiple tissue and tumor punches taken from formalin-fixed, paraffin-embedded blocks using a tissue cylinder with a diameter of 0.6 mm which were subsequently transferred into one recipient paraffin block using a homemade semiautomated tissue arrayer. Each patient had an average of 4 tumor punches included on the area, including 2 from the tumor center and 2 from the invasive front. Immunohistochemistry was performed for the tissue microarray according to the protocols described above for the Test Group. Protein expression was evaluated according to the scoring method described above by an experienced gastro-intestinal pathologist (E.K.).

**Table 1:** Characteristics of patients with mismatch repair-proficient colorectal cancer.

Features		Frequency n (%)	
		Test Group (n = 1197)	Validation Group (n = 209)
Age (yrs)	Mean, range	71 (30-96)	71•0, 35-93
Gender	Female	596 (50.0)	101 (50•5)
	Male	595 (50.0)	99 (49•5)
Tumor location	Left-sided	383 (32.5)	124 (62•0)
	Right-sided	343 (29.1)	48 (24•0)
	Rectum	451 (38.3)	28 (14•0)
Histologic subtype	Mucinous	89 (7.4)	25 (12•5)
	Non-mucinous	1108 (92.6)	175 (87•5)
pT classification	pT1-2	249 (21.4)	49 (24•5)
	pT3-4	916 (78.6)	151 (75•5)
pN classification	pN0	587 (51.4)	101 (50•5)
	pN1-2	555 (48.6)	99 (49•5)
Tumor grade	G1-2	1037 (89.1)	121 (75•7)
	G3	128 (11.0)	39 (24•4)
Vascular invasion	Absent	834 (71.5)	168 (81•9)
	Present	332 (28.5)	37 (18•1)
Local recurrence	Absent	208 (55.2)	-
	Present	169 (44.8)	
Distant metastasis	Absent	313 (80.9)	183 (90•6)
	Present	74 (19.1)	19 (9•4)
Post-operative therapy	None	289 (76.7)	72 (35•1)
	Treated	88 (23.3)	133 (64•9)
Median (months)	Rate (95%CI)	73.0 (63-85)	58.0 (48-65)

### Flow cytometry analysis on fresh clinical specimens

Ten freshly excised clinical specimens were collected and tumor fragments were minced and enzymatically digested in order to obtain single cell suspensions. Cells were surface stained with Fluorescein isothiocyanate (FITC)-labeled antibodies specific for CD4, CD16 or CD66b (BD Pharmingen, San Diego, CA) or T-cell-receptor (TCR)γδ (Ebioscience, La Jolla, CA) molecules and with APC-labeled antibodies specific for CD56 or Vα24-Jα18 TCR (BD Pharmingen). After washing, cells were fixed with 2% formaldehyde and subsequently permeabilized with 0.5% saponin prior to intracellular staining with PE-labelled TIA-1-specific antibodies (Immunotech, Marseille, France).

### Study Design

**Figure 1:**
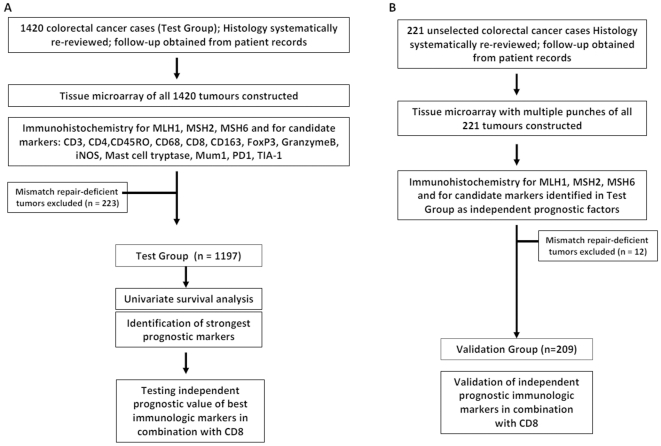
Overview of study design. (A) 1420 colorectal cancers were mounted onto tissue microarrays and immunostained for 13 protein markers and mismatch repair (MMR) proteins. MMR-deficient tumors were excluded. (B) 221 colorectal cancers were mounted into a multiple-punch tissue microarray and immunostained for independent prognostic factors identified in Test Group. MMR-deficient tumors were excluded from analysis and validation of prognostic effects carried out on the remaining 209 patients.

The study design is outlined in **Figure 1**. Briefly, after exclusion of MMR-deficient cases from the test cohort, univariate analysis and multivariable survival time models were assessed to identify the strongest independent prognostic protein markers and their combined value. The independent prognostic value was subsequently evaluated on MMR-proficient colorectal cancer patients from the Validation Group.

### Statistical Analysis

In order to avoid bias from dichotomizing protein immunoreactivity, analyses were performed by examining scores 0, 1, 2 and 3 [11]. Kaplan-Meier curves were used to assess the influence of protein expression on overall cancer-specific survival. Significance was assessed in univariate analysis with the log-rank test. Cox proportional-hazards models were used to test the simultaneous influence on overall cancer-specific survival of protein expression along with known prognostic factors and the assumption of proportional hazards was tested by evaluating the log(-log(survival)) versus log of survival time graphs. Rather than performing split-group analysis, all multivariable models were validated and 95%CI obtained through 200 bootstrapped replications of the data [12], [13]. All tests were two-sided. Missing variables were considered to be missing at random. No imputation was performed rather case-wise deletion was carried out when necessary. P-values are reported without adjustment for multiple corrections [14]. All analyses were performed with SAS V9.1 (The SAS Institute, Cary, NC, USA).

## Results

### Test Group

#### Cancer-specific survival analysis- Univariate (Figure 2)

More favourable survival time was observed for patients with higher numbers of CD3+ (p<0.001), CD4+ (p = 0.029), CD8+ (p<0.001), CD45RO+ (p = 0.048), FoxP3+ (p<0.001), GranzymeB+ (p<0.001), iNOS+ (p = 0.035), MUM+1 (p = 0.014), PD1+ (p = 0.034) and TIA-1+ TILs (p<0.001). Representative photomicrographs of these protein markers and immunoreactive cells are shown in Figure 3.

**Figure 2:**
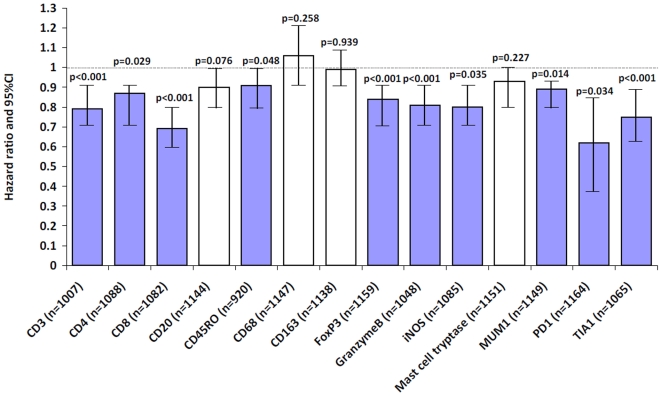
Bar graphs illustrating the hazard ratio and 95%CI for the prognostic effect of each biomarker. Highlighted markers are those showing a significant association with prognosis.

**Figure 3:**
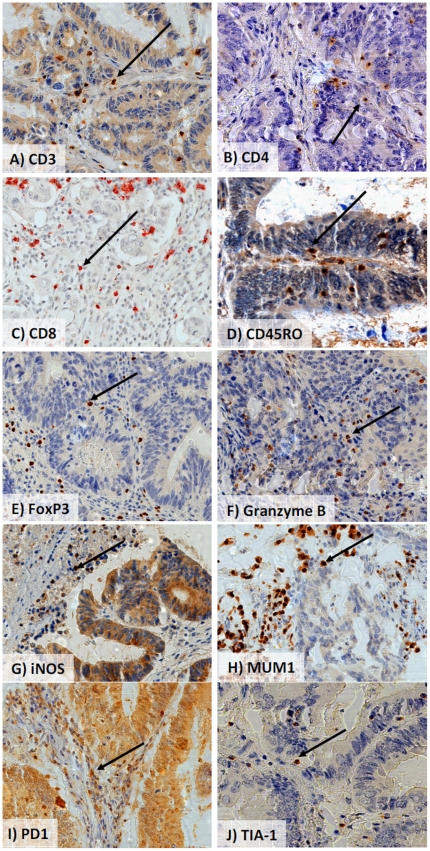
Representative immunostains for biomarkers with prognostic significance in the Test Group. A) CD3, B) CD4, C) CD8, D) CD45RO, E) FoxP3, F) Granzyme B, G) iNOS, H) MUM1, I) PD1, J) TIA-1.

#### Cancer-specific survival analysis- Multivariable

Significant markers were tested in two multivariable models. We first evaluated the prognostic effect of the markers adjusting for the effects of age at diagnosis, gender, pT, pN, tumor grade and vascular invasion. CD3 (n = 945; 434 deaths; p = 0.128), CD4 (n = 1026; 491 deaths; p = 0.463), CD45RO (n = 866; 401 deaths; p = 0.181), FoxP3 (n = 1091; 519 deaths; p = 0.185), GranzymeB (n = 987; 479 deaths; p = 0.091), iNOS (n = 1023; 493 deaths; p = 727), MUM1 (n = 1082; 516 deaths; p = 0.173), and PD1 (n = 1096; 523 deaths; p = 0.389) did not show an effect on survival time after adjusting for these established prognostic parameters. In contrast, CD8 (n = 1019; 489 deaths; p<0.001) and TIA-1 (n = 1005; 481 deaths; p<0.001) maintained their highly positive and significant impact on patient outcome.

In a second survival time model (**Table 2**) the effect of CD8 and TIA-1 was again tested this time along with patient age at diagnosis, gender, pT, pN, metastasis and adjuvant therapy. Results underline a highly significant positive effect of two markers: (1) CD8+ (n = 292; 75 deaths; HR: 0.66 (95%CI: 0.64-0.68) and (2) TIA-1+ (n = 290; 82 deaths; HR: 0.56 (95%CI: 0.5-0.6)).

**Table 2:** Multiple Cox regression analysis showing the independent prognostic effect of CD8, TIA-1 and of TIA-1 among CD8+ cases (all p-values <0.001).

	HR (95%CI)		
	CD8 alone	TIA-1 alone	Effect of TIA-1in CD8+ cases
Marker	0.66 (0.64-0.68)	0.57 (0.5-0.6)	0.89 (0.8-0.9)
Age (yrs)	1.03 (1.03-1.03)	1.03 (1.03-1.03)	1.05 (1.04-1.06)
Gender	0.72 (0.69-0.75)	0.64 (0.6-0.7)	0.56 (0.5-0.6)
pT	3.85 (3.51-4.23)	3.81 (3.5-4.1)	1.69 (4.1-5.3)
pN	3.01 (2.9-3.2)	2.24 (2.1-2.3)	2.56(2.4-2.8)
Metastasis	8.93 (8.5-9.4)	8.93 (8.5-9.4)	18.0 (16.6-20.0)
Post-operative therapy	1.45 (1.4-1.5)	1.88 (1.8-1.9)	2.12 (2.0-2.3)

#### Characterization of TIA-1-expressing cells

**Figure 4:**
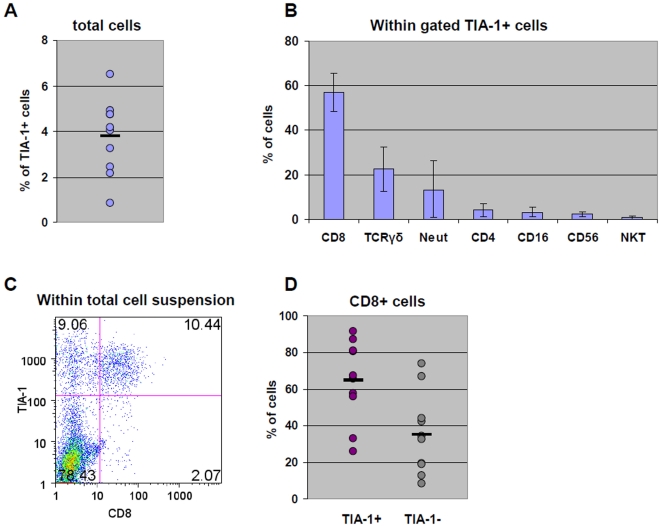
TIA-1 expression on TILs. Single cell suspensions obtained from freshly excised tumor specimens (n = 10) were surface stained with FITC- or APC-labeled antibodies specific for the indicated surface markers and intracellularly stained with PE-labeled anti-TIA-1 antibodies. A) Percentages of total TIA-1+ cells B) Percentages of CD8+ cells, TCRγδ cells, neutrophils (identified as CD66b+ cells), CD4+ cells, macrophages (identified as CD16+ CD56- cells), NK cells (CD16+ CD56+) and NKT cells (Vα24-Jα18 TCR+) observed within gated TIA-1+ cells. C) Representative dot-plot illustrating co-expression of TIA-1 and CD8 molecules within gated lymphocytes on total cells obtained from a clinical specimen. D) Percentages of TIA-1+ and TIA-1- cells within gated CD8+ cells.

Given that TIA-1 is hypothesized as a marker for activated CD8+ T-cells, we verified the coexpression of TIA-1 on CD8+ T cells by flow cytometry on tumor infiltrating lymphocytes (TILs) from 10 freshly excised clinical specimens. Percentages of total TIA-1+ cells ranged from 0.86 to 6.52 (mean: 3.7±1.6) (**Figure 4A**)**.** Costaining with markers specific for defined cell populations confirmed that the major population of TIA-1+ cells is represented by CD8+ T cells (57±8%) followed by TCRγδ cells (22±10%) and CD66b+ neutrophils (13±12%). In contrast negligible percentages of CD4+ T cells, macrophages (identified as CD16+ CD56- cells), NK cells (identified as CD56+ cells), and NK/T cells, (expressing Va24-Ja18 TCR), were observed (**Figure 4** **B, C**)**.** TIA-1 expression was detected on a majority of infiltrating CD8+ cells (64±22%), whereas the remaining fraction (35±22%) was TIA-1 negative (**Figure 4C, D**).

#### Phenotypic combinations of CD8/TIA-1

We next analyzed the phenotypic frequencies of the 1019 colorectal cancers with both evaluable CD8 and TIA-1 staining. Three major phenotypes were observed: tumors negative for both CD8 (Score 0) and TIA-1 (Score 0) (n = 223; 21.9%), tumors positive for CD8 but negative for TIA-1 (n = 366; 36%) and tumors positive for both CD8 and TIA-1 (n = 350; 34.3%). Only 80 tumors were negative for CD8 and positive for TIA-1 (7.8%). Due to these small numbers, the group of CD8 negative tumors was classified as a single subgroup irrespective of TIA-1 expression.

#### Cancer-specific survival analysis of CD8+ TILs stratified by TIA-1

**Figure 5:**
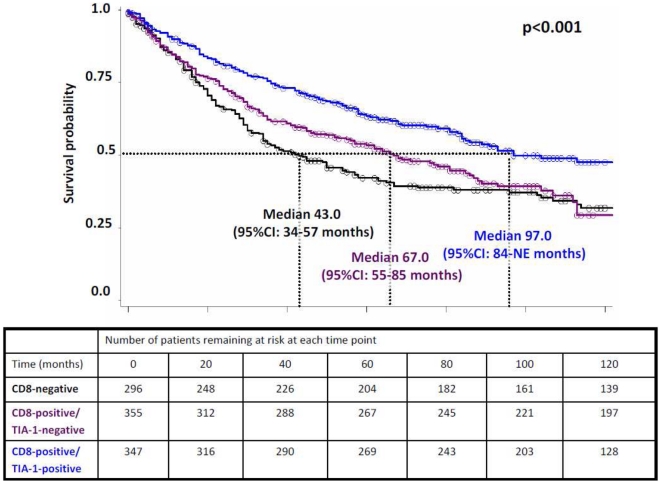
Kaplan-Meier survival curve shows the prognostic effect of CD8-negative, CD8+/TIA-1- and CD+8/TIA-1+ cells (Test Group).

Survival time differences were analyzed for the three CD8/TIA-1 phenotypes in 998 patients for whom survival time was also available. Overall, a significant difference in cancer-specific survival times was noted between the three groups with patients with higher amounts of CD8+/TIA-1+ TILs experiencing the most favourable outcome, followed by CD8+/TIA-1- cases and finally by CD8- patients (p<0.0001; **Figure 5**). We next performed multivariable analysis including the prognostic factors age, gender, pT, pN pM stage as well as post-operative therapy and determined the added benefit of stratifying patients with tumors with higher amounts of CD8+ TILs by TIA-1. Among patients with CD8-positive tumors, a relative risk of death of 0.89 (95%CI: 0.8-0.9) was observed for those with simultaneous TIA-1-positive tumors (p<0.001; **Table 2**). These results indicate that the addition of TIA-1 in cases of CD8-positivity improves the risk stratification for 35.6% (n = 355) of patients.

### Validation Group

An external validation set was analysed for CD8+ and TIA-1+ TILs. In this group of MMR-proficient colorectal cancer, only 7 patients had tumors with CD8+/TIA-1- TILs In univariable analysis, survival time differences for patients with CD8+/TIA-1+ TILs were significantly higher compared to patients with absence of CD8+ TILs (p<0.0001). Survival time differences between patients with different CD8/TIA-1 phenotypes were evaluated in multivariable analysis with pT stage, pN stage, pM and adjuvant therapy. Among patients with CD8-positive tumors, TIA-1 positivity led to a relative risk of 0.79 (95%CI: 0.7-0.9) compared to patients with TIA-1-negative cancers (p = 0.006).

## Discussion

The novel findings of this study on 1406 MMR-proficient colorectal cancer patients suggest first, that higher numbers of TIA-1+ TILs represent a favourable and independent prognostic parameter and second that in addition to CD8, TIA-1 improves the prognostic stratification of patients by 35%.

Although the prognostic significance of abundant CD8+ TILs has previously been established [3], [5], [15], [16], [17], this study goes one step further to identify the marker TIA-1 as a highly relevant prognostic parameter in colorectal cancer and particularly in tumors with marked cytotoxic CD8+ TILs. TIA-1 is a cytoplasmic granule-associated RNA binding protein reportedly expressed in cells with cytolytic potential, including 50-60% of CD8+ T-lymphocytes [18]. TIA-1 has been shown to be involved in Fas-mediated apoptosis in a variety of human malignancies, and to sensitize endothelial cells to pro-apoptotic stimuli while also enhancing NK cell cytotoxic activity [19], [20], [21]. Our results are in agreement with these findings. Here, we show that 64% of CD8+ TILs co-express TIA-1. Additionally, we identify a small population of CD8- but TIA-1+ tumor infiltrating cells representing populations of TCRγδ cells and neutrophils and to a lesser extent CD4+ T cells, macrophages, NK cells, and NK/T cells.

Investigations on the clinical and prognostic implications of TIA-1+ lymphocytes is mostly restricted to haematological malignancies with both favourable and unfavourable effects attributed to over-expression of this protein [22], [23]. Only a handful of studies have evaluated TIA-1 expression on TILs in colorectal cancer, relating up-regulation to apoptosis and an increased number of TIA-1+ TILs in MMR-deficient tumors [24], [25]. Interestingly, in our MMR-proficient cases, we could not find any correlation between higher numbers of TIA-1+ cells and tumor expression of the anti-apoptotic marker Bcl-2, pro-apoptotic markers Apoptosis Activating Factor-1 (APAF-1) and Mammalian Sterile20-like kinase 1 (MST-1), nor with p53 or the proliferation marker Ki67 (data not shown). However, we not only report that increased numbers of TIA-1+ TILs is strongly associated with an improved clinical outcome, but represent an independent prognostic factor, in test and validation study cohorts. Most importantly, our results indicate that the effect of TIA-1 on outcome is additive to CD8, a result which has implications for 35% of all patients. Hence, the significantly more favourable outcome of patients with CD8+/TIA-1+ TILs compared to cases with CD8+/TIA-1- TILs may be indicative of a more efficient immunosurveillance by TILs with an activated and highly cytotoxic potential.

Results of this study also identified several other types of TILs within the tumor center as having a positive impact on prognosis, for example FoxP3. The prognostic role of Tregs seems to vary significantly by tumor type possibly indicating differential roles of these cells in a tissue-dependent manner [26]–[28]. In colorectal cancer, most studies are in line with our findings indicating that a high number of FoxP3 Tregs is a favourable prognostic factor for disease-free and overall survival time [10], [29], [30]. However, this effect, as highlighted here, does not appear to be independent of CD8+.

Although our study may be limited by incomplete clinical information in some cases, we could still confirm the independent prognostic effect of TIA-1 and its modifying effect on CD8 in 500 patients with complete TNM and therapy information. The MMR status was analysed by expression of protein markers MLH1, MSH2 and MSH6 and only tumors with positivity in all three markers were included in this analysis. These markers as well as TIA-1 and CD8 are routinely used in diagnostic pathology, the latter two for T-cell lineage determination and subtyping of lymphomas thus supporting their value as reliable research tools as well [31], [32]. The use of TMAs often raises the concern that protein expression of heterogeneous tumors is not adequately represented using this technique. However, in daily diagnostic work, the occurrence of TILs is known to be quite homogeneous and while one tumor punch was sampled in the Test Group, the additional inclusion of a validation cohort with an average of 4 tumor punches per tumor sampled should minimize bias from possible tumor heterogeneity [33]. Furthermore, our study benefits from the use of test and validation cohorts, the latter representing an independent, external validation subset on which protein markers were assessed by multiple independent pathologists, thus further confirming the reproducibility of the CD8/TIA-1 scoring system and effect on outcome.

To summarize, TIA-1 is a robust prognostic immunological biomarker that contributes to clinical outcome in patients with MMR-proficient colorectal cancers independently of TNM stage and adjuvant therapy. The combined analysis of CD8/TIA-1 identifies a large subgroup of patients, who benefit from improved risk stratification, which should aid to tailor more individual clinical management for patients with colorectal cancer patient, while providing a new potential avenue of investigation for the development of targeted immunotherapy.
